# Altered Brain Activity in Patients With Comitant Strabismus Detected by Analysis of the Fractional Amplitude of Low-Frequency Fluctuation: A Resting-State Functional MRI Study

**DOI:** 10.3389/fnhum.2022.874703

**Published:** 2022-04-08

**Authors:** Meng-Yan Hu, Yi-Cong Pan, Li-Juan Zhang, Rong-Bin Liang, Qian-Min Ge, Hui-Ye Shu, Qiu-Yu Li, Chong-Gang Pei, Yi Shao

**Affiliations:** Department of Ophthalmology, Jiangxi Province Ocular Disease Clinical Research Center, The First Affiliated Hospital of Nanchang University, Nanchang, China

**Keywords:** fractional amplitude of low-frequency fluctuation, comitant strabismus, brain changes, depression and anxiety, resting-state functional magnetic resonance imaging

## Abstract

More and more studies showed that strabismus is not simply an ocular disease, but a neuro-ophthalmology disease. To analyze potential changes in brain activity and their relationship to behavioral performance in comitant strabismus patients and healthy controls. Our study recruited 28 patients with comitant strabismus and 28 people with matched weight, age range, and sex ratio as healthy controls. Using resting-state functional magnetic resonance imaging, we evaluated fALFF to compare spontaneous brain activity between comitant strabismus and healthy controls. We did hospital anxiety and depression scale questionnaires for these patients. We found significantly lower fALFF value in comitant strabismus patients compared with controls in the left frontal superior medial gyrus and the right middle cingulum. In the latter region, fALFF was significantly negatively correlated with the hospital anxiety and depression scale, as well as the duration of disease. Receiver operating characteristic curve analysis indicated that the fALFF method has clear potential for the diagnosis of comitant strabismus patients. These results revealed abnormal spontaneous activity in two brain regions of comitant strabismus patients, which may indicate underlying pathologic mechanisms and may help to advance clinical treatment.

## Introduction

Strabismus is an eye disorder characterized by vertical, horizontal or rotational deviation of one eye relative to the other, and can lead to binocular visual impairment and amblyopia, further result in neuropathology outcomes, such as depression and anxiety (Chia et al., 2010; Feng et al., 2015). More and more studies showed that strabismus is not simply an ocular disease, but a neuro-ophthalmology disease (Jackson et al., 2006; Cumurcu et al., 2011; Alpak et al., 2014). According to a meta-analysis, the prevalence of strabismus was about 2.0%, regardless of age or sexual difference (Hashemi et al., 2019). In another study conducted in Eastern China, strabismus prevalence was 5.65% (320 out of 5,667) in preschool students aged 36-72 months, among the 320 strabismus students, 302 were diagnosed as comitant strabismus (CS) (Chen et al., 2016). Comitant strabismus is the most common type of strabismus characterized by constant angle of strabismus (deviation) in all directions of gaze (Robaei et al., 2006). With either eye used for fixation, the deviation angle remains unchanged. The etiology and pathophysiology of CS is complex and remains unclear. Several risk factors may have contributed to the onset or progress of CS, such as family history, systemic disease, genetic syndrome, dysfunction of extraocular muscles, as well as neuromuscular imbalance (Gunton et al., 2015). Stereopsis impairment and abnormal eye position are the main clinical manifestations of strabismus in adults. Meanwhile, CS children may suffer more severe complication. In addition to disabling diplopia and cosmetic consequence, it may further impair learning ability and result in psychosocial problem (Jackson et al., 2006; Chia et al., 2010). Other studies also showed psychiatric problems in CS patients, such as anxiety and depression (Cumurcu et al., 2011; Alpak et al., 2014). At present, surgical treatment is the most effective method of strabismus correction, which may be important not only for visual correction but also to improve cosmetic and social anxiety, especially in some groups of patients, such as graduates (Estes et al., 2020).

Previous studies showed brain changes in depressed patients, such as enhanced default mode network connectivity with ventral striatum, lower cortical thickness in the left frontal brain regions (Fonseka et al., 2016; Hwang et al., 2016). Another study found declined insular volume in patients with social anxiety (Kawaguchi et al., 2016). As mentioned above, depression and anxiety disorders were also revealed in CS patients, thus brain abnormalities may also exist in CS patients.

In fact, animal studies on strabismus have already revealed functional alterations in the visual cortex and connection changes between brain regions. Studies on strabismic monkeys demonstrated both structural (neuron loss) and functional (reduced metabolic activity) alterations in the primary visual cortex (Crawford and von Noorden, 1979; Adams et al., 2013). Moreover, Schmidt and Lowel (2006) made strabismus animal model via cats, they found that functional maps in area 17 were affected by experience-dependent manipulations, while those in area 18 remain still (Schmidt and Lowel, 2006).

With the development in magnetic resonance imaging technique, we are able to evaluate the human brain changes functionally and anatomically in resting-state (Biswal, 2012). The resting-state functional magnetic resonance imaging (rs-fMRI) method can provide important information about brain activity and it is widely used to investigate spontaneous functional magnetic resonance imaging signals (Zang et al., 2004; Wang et al., 2012; Song et al., 2021). Compared to task-based fMRI, rs-fMRI analyses signal without any specific task or an input, thus patients who have difficulty in accomplishing the task, i.e., pediatric patients, can still undergo rs-fMRI (Smitha et al., 2017).

The amplitude of low-frequency fluctuations (ALFF) is a common rs-fMRI technique used to investigate spontaneous brain activity at rest via measuring the blood-oxygen level-dependent (BOLD) signals (Logothetis et al., 2001; Zuo et al., 2010; Huang et al., 2015; Shao et al., 2015). ALFF reflects the amplitude of signal fluctuations in a single time series per voxel, not unlike the concepts of “power” or “energy” used in electroencephalogram studies. Statistically, it reflects the mean deviation, standard deviation or variance of signals in a given frequency band, which are all relative values. In contrast, fractional ALFF (fALFF) is a normalized parameter that reflects the contribution of specific low-frequency oscillations relative to the entire frequency range. It is less sensitive to physiological noise than that in ALFF method, indicating that fALFF may detect abnormal brain activities with higher sensitivity and specificity (Zou et al., 2008). The fALFF method has already been applied to study potential neuropathological mechanisms of ophthalmologic diseases such as monocular blindness, different kinds of glaucoma (Li et al., 2014, 2020; Fang et al., 2020; Wang et al., 2021), as well as neurogenic diseases like narcolepsy, idiopathic epilepsy and Parkinson’s disease (Qiao and Niu, 2017; Tang et al., 2017; Xiao et al., 2018). But to our knowledge it has not been used in CS. In this study, we used the fALFF method to investigate the relationship between CS and brain activity.

## Materials and Methods

### Subjects

Twenty-eight patients with comitant strabismus and another twenty-eight healthy controls were recruited from the First Affiliated Hospital of Nanchang University. The diagnostic criteria of comitant strabismus were: (1) congenital strabismus; (2) stereovision defect (no visual fusion); (3) in the case of alternating strabismus, the strabismus angles were equal; (4) best corrected visual acuities > 1.0 in both eyes.

Twenty-eight healthy controls (HCs) were recruited, matched in terms of age range, sex ratio and education level and with uncorrected or best corrected visual acuity of 1.0 or better.

Participants who met any of the following conditions were excluded from the study: (1) acquired or incomitant strabismus, concealed oblique, and amblyopia, as well as diplopia; (2) history of intraocular or extraocular eye surgery; (3) history of eye diseases (infection, inflammation, ischemic diseases); (4) history of refractive error (myopia higher than −1.5D, hyperopes or anisometropes); (5) history of mental health disorders (such as anxiety disorder, obsessive-compulsive disorder, depression or schizophrenia), diabetes, cerebral infarction or cardiovascular diseases; (6) history of addictions (alcohol and/or drugs); (7) abnormality of brain parenchyma shown by MRI; 8) contraindication for MRI scanning.

All research methods were approved by the committee of the medical ethics of the First Affiliated Hospital of Nanchang University and were in accordance with the 1964 Helsinki declaration and its later amendments or comparable ethical standards. All subjects were explained the purpose, method, potential risks and signed an informed consent form.

### Administration of Hospital Anxiety and Depression Scale

The Hospital Anxiety and Depression Scale (HADS) is a simple yet reliable tool for assessing anxiety and depression (Snaith, 2003). For participants who were illiterate or were unable to read due to visual impairment, an investigator read the questionnaire aloud. In all participants, an investigator verbally explained the purpose of the questionnaire and its confidential nature.

### Magnetic Resonance Imaging Scanning

Magnetic resonance imaging scanning was performed using a 3T MR scanner (Trio, Siemens, Munich, Germany). The subjects were in the supine position, wearing earplugs to reduce noise during scanning, and were required to stay awake with eye closed and maintain quiet breathing throughout the scanning period of 15 minutes. The range of head movement during scanning was < 3mm, and the head rotation range was <2.5°. The structural images were obtained using single-shot gradient-recalled echo planar imaging sequence with following parameters: repetition time/echo time = 1900/2.26 ms, flip angle = 9°, field of view = 250 × 250 mm, matrix = 256 × 256, slice thickness/gap = 1.0/0.5 mm, images = 176. Besides, each participant underwent three-dimensional metamorphic gradient echo pulse planar imaging with parameters as follows: repetition time/echo time = 2000/40 ms, flip angle = 90°, field of view = 220 × 220 mm, matrix = 64 × 64, slice thickness/gap = 4.0/1.0 mm, images = 240. 30 axial slices were obtained at each time point. The whole scanning time was 8 minutes and no substantial lesions of the brain were found.

### Rs-FMRI Data Processing

For data acquisition and processing, MRIcron^1^ was employed to classify functional data and eliminate incomplete data. Statistical Parametric Mapping (SPM8)^2^ was used to preprocess rs-fMRI images. The processing protocols were as follows: (1) the original data were converted to Neuroimaging Informatics Technology Initiative (NIFTI) format; (2) The first 10 functional images were removed to balance the signal; (3) slice timing and head motion correction; (4) normalization of rs-fMRI images to the standard Montreal Institute of Neurology (MNI) templates and resampled to 3 × 3 × 3mm voxels; (5) image smoothing with a 6-mm full-width-half-maximum (FWHM) Gaussian to reduce spatial noise; (6) regression of covariates including six head movement parameters, mean framewise displacement, and average signals from white matter, cerebrospinal fluid, and global brain activity; (7) removal of linear trends.

### fALFF Analysis

The fALFF method was used to analyze the fMRI data. The Resting-State fMRI Data Analysis Toolkit (REST)^3^ was used to convert time series data into the frequency domain and calculate the power spectrum. The fALFF value was calculated as the ratio of the low-frequency range (0.01–0.08 Hz) to the power spectrum of the entire frequency range (0–0.25 Hz). In addition, the fALFF value was normalized to the whole-brain mean fALFF to reduce inter-subject variability, and a band-pass filtering of 0.01-0.08 Hz was used to minimize deviation from heartbeats and respiratory rhythm.

### Statistical Analysis

Brain areas with significant changes in frequency or overall fALFF were selected as regions of interest (ROIs). Two-sample *t*-tests were used to compare fALFF values at the ROIs between the CS and HC groups with sex and age as covariates to control for these factors. Receiver operating characteristic (ROC) curves were also used to assess sensitivity of the discrimination between mean fALFF values in the two groups. In addition, Pearson’s correlation was used to assess the relationship between fALFF and both disease duration and HADS score. Analyses were conducted using SPSS version 13.0 statistical software for Windows (SPSS, IBM Corp., United States), and statistical figures were generated by GraphPad Prism 8 software (the GraphPad Software, Inc. La Jolla, CA, United States). * meant *P* value < 0.05 and was considered statistically significant in all cases.

## Results

### Demographics and Visual Measurements

No significant between-group differences were found (Table 1) in sex (*p* > 0.99), age (*p* = 0.324), weight (*p* = 0.585), handedness (*p* > 0.99), best-corrected VA-DE (*p* = 0.256), or best-corrected VA-FE (*p* = 0.212) between CS and HCs.

**TABLE 1:** The conditions of participants included in the study.

Condition	CS	HCs,	*t*-value	*P*-value*
Male/female	18/10	18/10	N/A	> 0.99
Age (years)	17.39 ± 3.56	18.68 ± 2.56	−1.285	0.365
Weight (kg)	56.76 ± 3.66	60.59 ± 3.13	−0.674	0.576
Handedness	28R	28R	N/A	> 0.99
Best-corrected VA-DE	1.05 ± 0.10	1.15 ± 0.10	1.645	0.243
Best-corrected VA-FE	1.05 ± 0.15	1.15 ± 0.10	1.714	0.252
Duration of CTR (days)	17.39 ± 3.56	N/A	N/A	N/A
Esotropia/exotropia	10/18	N/A	N/A	N/A
Angle of strabismus (PD)	40.25 ± 11.05	N/A	N/A	N/A

*Independent t-tests comparing two groups (P < 0.05 represented statistically significant differences). CS, comitant strabismus; HCs, healthy controls; N/A, not applicable; PD, prism diopter; VA, visual acuity; DE, dominant eye; FE, fellow eye.*

### RsfMRI-FALFF Results and Brain Regions

**FIGURE 1:**
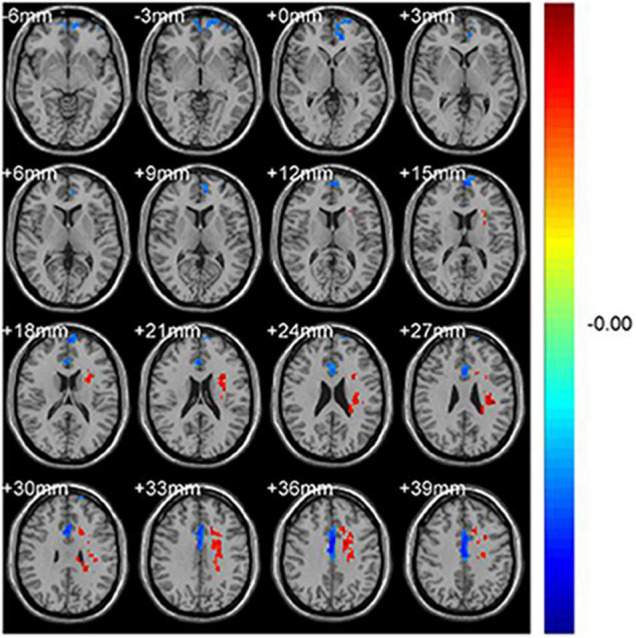
Significant differences in fALFF values between the CS group and HCs. The brain regions with different fALFF values are the Frontal_Sup_Medial_L and the Cingulum_Mid_R. The red areas denote higher fALFF brain regions and blue areas denote lower fALFF brain regions.

**FIGURE 2:**
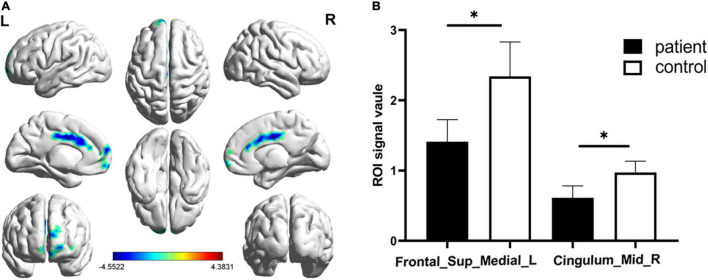
Significantly altered fALFF values in patients with CS compared with HCs. The significantly altered regions were located in the Frontal_Sup_Medial_L and the Cingulum_Mid_R. Red and blue areas denote regions with higher and lower fALFF, respectively **(A)**. Statistical thresholds were set at P, 0.01 (two sample *t*-test, corrected for false discovery rate) with a minimum cluster size of 75 voxels (**B**, *p* = 0.012 and *p* = 0.039 respectively). *p < 0.05.

Significantly lower fALFF values were found in the CS compared with HC group in the Frontal_Sup_Medial_L and the Cingulum_Mid_R brain regions (*p* = 0.012 and *p* = 0.039, respectively) (Figures 1, 2). Corresponding voxels and coordinate are demonstrated (Table 2).

**TABLE 2:** Brain regions with significant differences in brain activities between CS and HCs.

Brain areas	MNI coordinates			BA	Number of voxels	*T*- value
	
	X	Y	Z			
HC > patient						
Frontal Sup Medial L	−12	48	0	31	156	−3.6533
Cingulum Mid R	3	−9	36	34	205	−4.5522

*The statistical threshold was set at voxel with p < 0.01 for multiple comparisons using Gaussian random field corrected. MNI, Montreal Neurological Institute; BA, brodmann area.*

### ROC Curve Analysis

**FIGURE 3:**
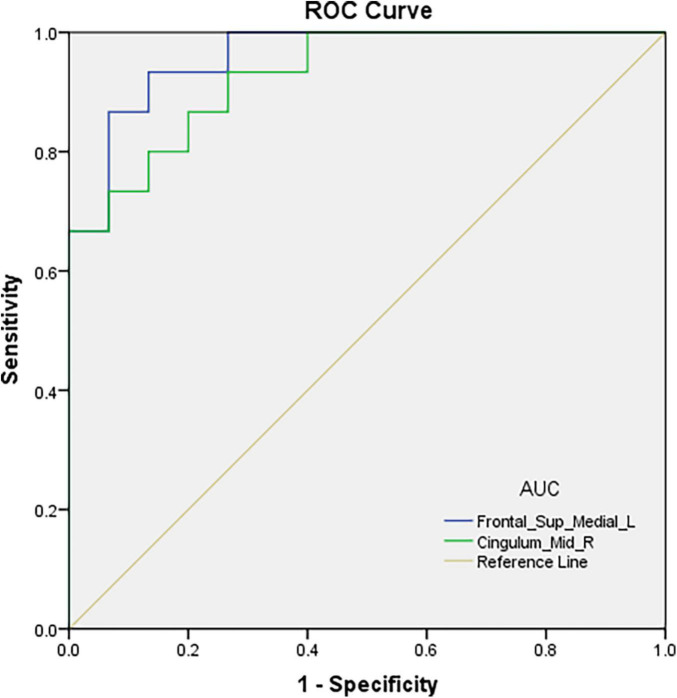
ROC curve analysis of the fALFF values for altered brain regions. The area under the ROC curve were 0.960, (*p* < 0.001; 95% CI: 0.899-1.000) for Frontal_Sup_Media l_L, and 0.929, (*p* < 0.001; 95% CI: 0.843-1.000) for Cingulum_Mid_R.

ROC curve analysis was conducted using fALFF values of the two brain regions found to differ between the groups. The areas under the ROC curves were 0.960 (*p* < 0.001; 95% CI: 0.899-1.000) for Frontal_Sup_Medial_L and 0.929 (*p* < 0.001; 95% CI: 0.843-1.000) for the Cingulum_Mid_R (Figure 3).

### Correlation Analysis of the fALFF Values and Clinical Measurements in Comitant Strabismus Patients

**FIGURE 4:**
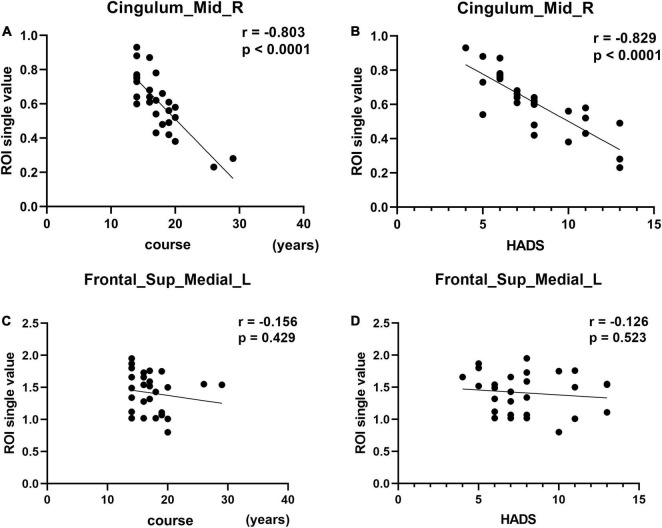
Correlation between the fALLF values of the two brain regions and course and HADS. In the CS group, the fALFF value of the Cingulum_Mid_R showed a negative correlation with the course (**A**, *r* = –0.803, *p* < 0.0001). The fALFF value of the Cingulum_Mid_R showed a negative correlation with the HADS (**B**, *r* = –0.829, *p* < 0.0001). The fALFF values of Frontal_Sup_Medial_L showed no correlation with the course of the disease (**C**, *r* = –0.156, *p* = 0.429) or HADS score (**D**, *r* = –0.126, *p* = 0.523).

Mean Cingulum_Mid_R fALFF values in CS patients were negatively correlated with duration of the disease (*r* = −0.803, *p* < 0.0001) and with HADS score (*r* = −0.829, *p* < 0.0001) (Figures 4A,B). While in the Frontal_Sup_Medial_L, the fALFF values showed no correlation with duration of the disease (*r* = −0.156, *p* = 0.429) or HADS score (*r* = −0.126, *p* = 0.523) (Figures 4C,D).

## Discussion

The fALFF method can provide information about spontaneous brain activity, is considered to represent the process of spontaneity as well as function, and has been successfully applied in patients with ophthalmic and neurogenic diseases (Table 3). To our knowledge, however, this method has not previously been used in patients with comitant strabismus. In the present study, compared with healthy controls, significantly lower fALFF values were found in two brain regions, the left frontal lobe (Frontal_Sup_Medial_L) and the right cingulum (Cingulum_Mid_R), in patients with comitant strabismus. In addition, correlation analysis showed significant negative correlation between the fALFF values of the Cingulum_Mid_R and the duration of CS, as well as HADS score.

**TABLE 3:** fALFF method applied in ophthalmologic and neurogenic disease.

Ophthalmologic disease	Authors and Years	Disease
	Li et al., 2014, 2020; Fang et al., 2020; Wang et al., 2021	Monocular Blindness,
		POAG
		normal-tension glaucoma PACG
	Qiao and Niu, 2017; Tang et al., 2017; Xiao et al., 2018	Narcolepsy Idiopathic epilepsy
Neurogenic disease		
		Parkinson’s disease

While the fALFF method has not previously been used to assess spontaneous brain activities in patients with comitant strabismus, fMRI has been used with other analysis methods in research on comitant strabismus. Huang et al. used regional homogeneity (ReHo) methods to analyze brain activity in comitant strabismus and found significantly increased ReHo values in several brain areas, including the cingulate gyrus (Huang et al., 2016). In another ALFF study, congenital comitant strabismus patients showed significantly higher ALFF values in some brain regions, while lower values were found in others, such as the frontal gyrus (Tan et al., 2016). Previous research by our group measured degree centrality (DC) values in comitant exotropia strabismus patients and found contrasting changes in different brain regions, such as increased DC values in the bilateral anterior cingulate, and decreased values in part of the frontal gyrus (Tan et al., 2018). There are still similar fMRI studies using different methods, such as voxel-based morphometry study,(Ouyang et al., 2017) and pseudo-continuous arterial spin labeling study (Huang et al., 2018), which we may not discuss in detail here. These findings are in broad agreement with those of the present study, with abnormal activity in some brain regions of patients with comitant strabismus.

The frontal lobe is a large part of the brain comprising four subcortical circuits. Lesions in each circuit manifest different disorders, such as voluntary movement, behavioral status, and mental activity disorders (Krudop and Pijnenburg, 2015). Besides, some part of the frontal lobe is important in integrating visual function, the frontal eye field (FEF) is located in the frontal cortex and has the function of triggering eye movements and influencing their accuracy or latency. Previous studies in monkeys have found that the FEF may play an important role in controlling eye movements (Kovelman et al., 2015). In addition, research has shown that the left middle frontal gyrus is associated with strabismus and amblyopia (Thompson et al., 1996). Specifically, lower ALFF values were in the left middle frontal gyrus in strabismic amblyopia than in controls, suggesting abnormal FEF function in this condition. Three brain regions including the Frontal_Sup_Medial_L have been identified as functional candidate hubs in the process of network information transmission (Min et al., 2018). Our finding of significantly lower fALFF value in left Frontal Sup Medial region in comitant strabismus may therefore suggest abnormal network transmission in this condition.

The cingulate cortex, as an important part of the limbic system, has three major divisions. Each division is in charge of different functions: the anterior for emotion states such as depression and anxiety, the middle for decision making and response selection, and the posterior for visuospatial orientation (Vogt, 2019). Shinoura et al. (2013) found improved visual memory after tumorectomy, along with aggravating anxiety disorder. Since the tumors were located just above the right dorsal anterior cingulate cortex (ACC), they speculated the improvement mainly came from the compression of the right dorsal ACC. In another ReHo study by our group, we found increased ReHo values in cingulate cortex in patients with strabismus and amblyopia (SA), indicating that cingulate gyrus played a compensatory role in the progress of SA (Huang et al., 2016). Meanwhile, contradictory to the studies mentioned above, our present study found reduced fALFF values in the Cingulum_Mid_R of patients with comitant strabismus. Since patients in our study all have a normal best-corrected visual acuity (BCVA), which means amblyopia patients were excluded in the study, subject differences may explain the contradiction. Further studies with more grouped patients are needed to clarify our findings. The alteration of these two brain regions and its potential impact were summarized as follow (Table 4).

**TABLE 4:** Brain regions alternation and its potential impact.

Brain regions	Experimental result	Brain function	Anticipated results
Frontal_Sup_Medial_L	CS < HC.	Information transmission	eye movements
Cingulum_Mid_R.	CS < HC	Executive function, attention and memory	depression, pain, and anxiety

A negative correlation was also found between fALFF values of this region and the HADS score in patients with CS. As is well known, emotion plays an important role in most diseases, and as outlined above this brain region is related to depression, pain, and anxiety. On this basis, we hypothesized that patients with CS have anxiety and depression, and this was confirmed by HADS score analysis.

As mentioned above, animal experiments on strabismus displayed primary visual cortex change (Crawford and von Noorden, 1979; Adams et al., 2013). While in our present study, no significant difference was found in primary visual cortex. Since patients in our study all have a normal best-corrected visual acuity (BCVA), which means amblyopia patients were excluded in the study, we thought this may partially explain why no difference was found in primary visual cortex.

**FIGURE 5:**
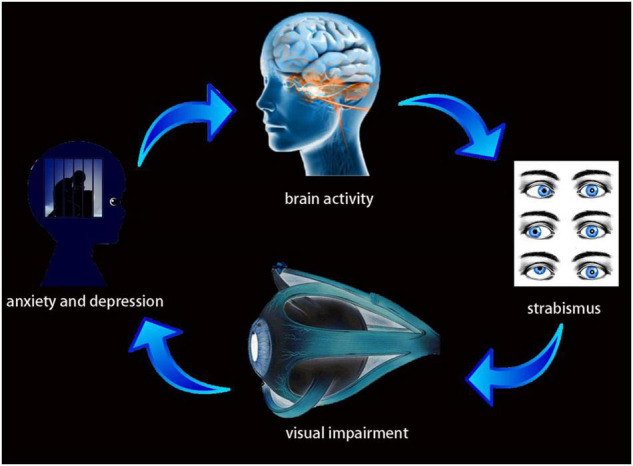
Relationship between MRI images and clinical manifestations in CS. Strabismus results from extraocular muscle dysfunction, leads to visual impairment, further result in anxiety and depression and alterations in brain activity.

Some previous anatomical studies have shown a relationship between the Frontal_Sup_Medial_L and the Cingulum_Mid_R (Bubb et al., 2018; Xiao et al., 2018). In the present study, activity in both of these regions was reduced in patients with comitant strabismus. Their relationship suggests the possibility that the frontal lobe injury affected the cingulate gyrus through fibrous connections, causing secondary changes in the latter, and thus triggering symptoms of anxiety and depression. We further speculated that comitant strabismus, resulting from extraocular muscle dysfunction, leads to visual impairment, further contributing to anxiety and depression and alterations in brain activity (Figure 5).

This study has some limitations. First, the sample size was not sufficiently large to subdivide patients into groups representing levels of visual acuity, strabismus angle, periods of disease development, and other variables. Larger sample rsfMRI studies may help better understand the explicit changes in brain activity and CS at different levels of severity or at different categories. Second, strabismus may cause psychological distress related to cosmesis, and this may have some impact on brain activity. Therefore, psychological factors should be considered to exclude the impact of emotional state. Finally, our study only showed microstructural changes in many brain regions in CS patients. Whether brain activity changes are secondary to strabismus or are the cause of strabismus remains unknown. In the future, larger, more in depth studies may help to address this question.

## Conclusion

In conclusion, our study found significant abnormalities in two brain regions (Frontal_Sup_Medial_L and Cingulum_Mid_R) in patients with comitant strabismus. ROC analyses indicated high accuracy of the fALFF method for diagnosing CS patients. Correlation analyses demonstrated that changes in Cingulum_Mid_R might help the severity of CS and predicted the incidence of depression in the CS patients. In the future, further investigations are needed to form a comprehensive understanding of the neuropathological mechanisms of comitant strabismus.

## Data Availability Statement

The raw data supporting the conclusions of this article will be made available by the authors, without undue reservation.

## Ethics Statement

The studies involving human participants were reviewed and approved by the committee of the medical ethics of the First Affiliated Hospital of Nanchang University. Written informed consent to participate in this study was provided by the participants’ legal guardian/next of kin.

## Author Contributions

M-YH, YS, and C-GP designed the current study. Y-CP and L-JZ recruited healthy controls. R-BL and Q-MG performed MRI scanning. H-YS and Q-YL collected and analyzed the data. M-YH wrote the manuscript. All the authors read and approved the final manuscript.

## Conflict of Interest

The authors declare that the research was conducted in the absence of any commercial or financial relationships that could be construed as a potential conflict of interest.

## Publisher’s Note

All claims expressed in this article are solely those of the authors and do not necessarily represent those of their affiliated organizations, or those of the publisher, the editors and the reviewers. Any product that may be evaluated in this article, or claim that may be made by its manufacturer, is not guaranteed or endorsed by the publisher.

## Footnotes

1
http://www.nitrc.org/projects/mricron

2
http://www.fil.ion.ucl.ac.uk/spm

3
http://restfmri.net/forum/REST

## Supplementary Material

Supplementary Figure 1 HADS value between CS patients and healthy controls. HCs showed significantly lower HADS scores than that of CS (*p* < 0.0001).
