# Supplementary figures and images for: Altered Brain Activity in Patients With Comitant Strabismus Detected by Analysis of the Fractional Amplitude of Low-Frequency Fluctuation: A Resting-State Functional MRI Study
Source: Front Hum Neurosci. 2022 Apr 8;16:874703. doi: 10.3389/fnhum.2022.874703 (PMC9027334; doi:10.3389/fnhum.2022.874703)

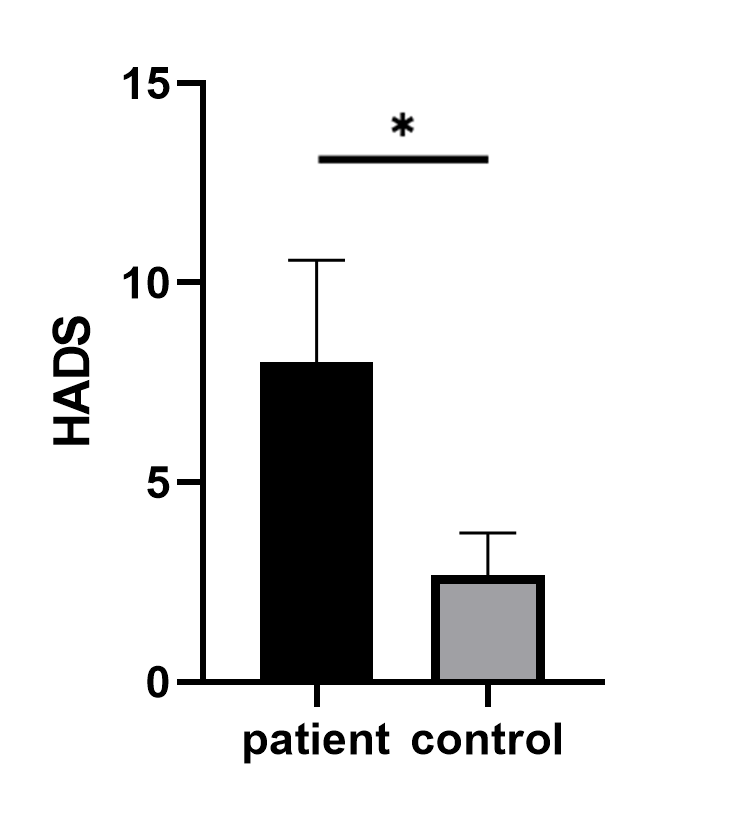

Supplement: Supplementary Figure 1 — HADS value between CS patients and healthy controls. HCs showed significantly lower HADS scores than that of CS (p < 0.0001). [file Image_1.TIF]
